# Mobile applications in radiation oncology—current choices and future potentials

**DOI:** 10.1007/s00066-023-02048-y

**Published:** 2023-02-21

**Authors:** Stefan Janssen, Rami A. El Shafie, Arne M. Ruder, Daniel Buergy, Davide Scafa, Frank A. Giordano, Nils H. Nicolay, Marco M. E. Vogel, Stephanie E. Combs, Fabian B. Fahlbusch, Dirk Rades, Lukas Käsmann

**Affiliations:** 1 Department of Radiation Oncology, University of Lübeck, Lübeck, Germany; 2 Private Practice of Radiation Oncology, Hannover, Germany; 3 Clinic of Radiotherapy and Radiation Oncology, University Medical Center Göttingen, Göttingen, Germany; 4 Department of Radiation Oncology, Heidelberg University Hospital, Heidelberg, Germany; 5 Department of Radiation Oncology, University Medical Center Mannheim, University of Heidelberg, Mannheim, Germany; 6 Department of Radiation Oncology, University Medical Center Bonn (UKB), Bonn, Germany; 7 Department of Radiation Oncology, University of Leipzig Medical Center, Leipzig, Germany; 8 Department of Radiation Oncology, University Hospital Klinikum rechts der Isar, Technical University of Munich (TUM), Ismaninger Str. 22, 81675 Munich, Germany; 9 Institute for Radiation Medicine (IRM), Department of Radiation Sciences (DRS), Helmholtz Zentrum München, Neuherberg, Germany; 10 Department of Pediatrics and Adolescent Medicine, Neonatology and Pediatric Intensive Care, Friedrich-Alexander-University of Erlangen-Nürnberg, Erlangen, Germany; 11 Department of Radiation Oncology, University Hospital, LMU Munich, Marchioninistr. 15, 81377 Munich, Germany; 12 Partner Site Munich, German Cancer Consortium (DKTK), Munich, Germany

**Keywords:** Electronic patient reported outcome (ePROs), Electronic health (e-health), Smart phone apps, Radiotherapy, Digitalization

## Abstract

**Purpose:**

To review existing scientific literature on mobile applications (apps) in the field of radiation oncology and to evaluate characteristics of commercially available apps across different platforms.

**Methods:**

A systematic review of the literature for publications presenting apps in the field of radiation oncology was carried out using the PubMed database, Cochrane library, Google Scholar, and annual meetings of major radiation oncology societies. Additionally, the two major marketplaces for apps, App Store and Play Store, were searched for available radiation oncology apps for patients and health care professionals (HCP).

**Results:**

A total of 38 original publications which met the inclusion criteria were identified. Within those publications, 32 apps were developed for patients and 6 for HCP. The vast majority of patient apps focused on documenting electronic patient-reported outcomes (ePROs). In the two major marketplaces, 26 apps were found, mainly supporting HCP with dose calculations.

**Conclusion:**

Apps used in (and for) scientific research in radiation oncology are rarely available for patients and HCP in common marketplaces.

## Introduction

Smartphones have revolutionized people’s lives, including the way they seek medical information [1]. In 2020, 78% of the worldwide population was in possession of a smartphone and the forecast predicts a further increase in the future [2]. There is a wide variety of health care applications (apps) for smartphones available. The top two categories are wellness management/fitness and disease management apps, whereas other categories include self-diagnosis, medication reminders, and electronic patient portal apps [1]. The World Health Organization (WHO) classifies such tools under the label mHealth or eHealth, and defines them as “medical and public health practice supported by mobile devices, such as mobile phones, patient monitoring devices, personal digital assistants, and other wireless devices” [3].

Cancer is a leading cause of disability and mortality worldwide [4]. Accordingly, a variety of cancer-focused apps exist and the number of research articles that study the use of apps in the field of general/clinical oncology is increasing [5]. The majority of health care professionals (HCP) are in favor of the use of oncological apps by patients according to Kessel et al. [6]. Moreover, the acceptance of mobile apps for the surveillance and follow-up of cancer patients undergoing radiotherapy is high [7, 8]. Indeed, there are several advantages like monitoring patient-reported outcomes (ePRO) and education of patients to increase compliance and optimize the HCP interaction. In addition, these applications can have positive psychologic effects by empowering patients, e.g., to track treatment, monitor side effects, or to schedule follow-up appointments [9].

Despite the large selection of apps in the field of oncology in general and the resonance in the scientific literature, little is known about their particular impact and use in the field of radiation oncology. Therefore, this review aims to summarize data on the scientific reception of apps in radiation oncology in the literature and to gain information on currently available apps for patients and HCP online.

## Methods

A systematic review of the literature in the PubMed database and Cochrane Library was performed in accordance with the Preferred Reporting Items for Systematic Reviews and Meta-Analyses (PRISMA) [10] in August 2022 using the terms (“radiotherapy” OR “radiation oncology” OR “radio-oncology”) AND (“smartphone app” OR “mobile application” OR “app”).

To improve the retrieval rate of studies, the reference sections of eligible articles were additionally screened. Moreover, Google Scholar was searched using the identical search terms. To further enhance search output, a manual search was conducted within the table of contents of annual meetings of the European Society for Radiotherapy and Oncology (ESTRO), the German Society of Radiation Oncology (DEGRO), and the American Society of Radiation Oncology (ASTRO), including oral and (e-) poster contributions (past 10 years). Furthermore, ClinicalTrials.gov was searched using the same search terms to detect ongoing trials (search field: “all studies” and “other terms”).

Inclusion and exclusion criteria were defined before the search. Inclusion criteria were as follows:

1. Original articles in English or German language.
2. Presentation of a mobile app for HCP working in the field of radiation oncology or patients being treated with radiotherapy (RT; in cases of mixed cohorts, the majority of patients had to be involved in RT; in cases of a concomitant use of web-based tools and smartphone apps, e.g., for ePRO collection, the majority had to be smartphone apps).

Exclusion criteria were:

1. Review articles.
2. Surveys.
3. Apps aimed at general/clinical oncology, without a focus on RT.

**Fig. 1:**
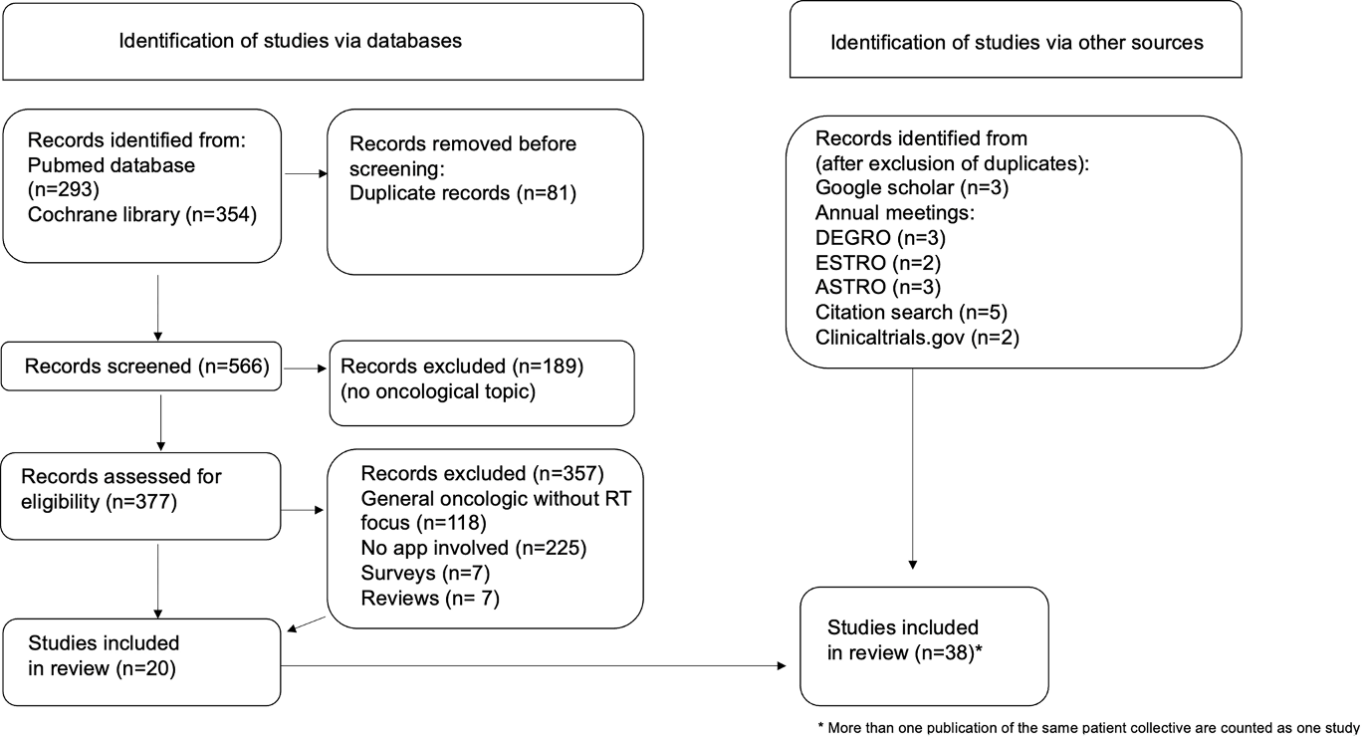
PRISMA flowchart for the search of scientific literature. *RT* radiotherapy

The respective PRISMA flowchart is given in Fig. 1.

**Fig. 2:**
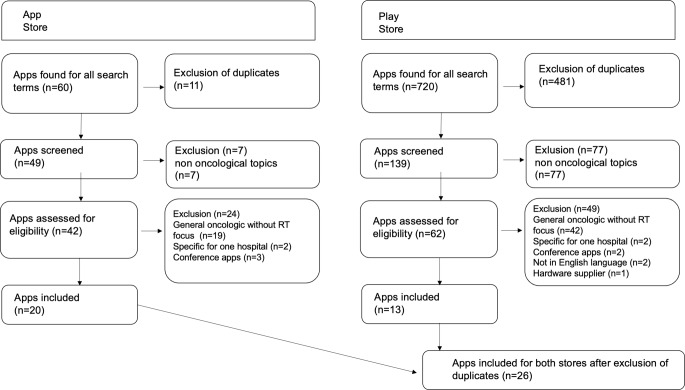
PRISMA flowchart for search in two major app stores. *RT* radiotherapy

Concomitantly, a search was conducted in the two major app stores to find commercially available smartphone apps. These two app stores were the Play Store (Android, Google; https://play.google.com/store) and App Store (iOS, Apple; https://www.apple.com/de/app-store). Search terms were “radiotherapy,” “radiation oncology,” and “radiation therapy.” The search was carried out in August 2022 independently by two authors (S.J. and L.K.) using an iPhone 13 and a Samsung Galaxy S10. The respective PRISMA flowchart is given in Fig. 2.

## Results

### Review of scientific literature

In total, 38 publications meeting the inclusion criteria were found (Table 1, 2 and 3). Different publications by the same study group concerning the same or mostly the same study cohort (e.g., study protocol, publication of preliminary and final results) were merged. Publications were categorized mainly in two different categories: apps developed for HCP (6 of 38, 16%) and apps developed for patients receiving RT or radiochemotherapy (RCT; 32 of 38, 84%). The latter publications were subdivided according to the patients’ underlying primary tumors: a mixed cohort of cancer patients receiving RT or RCT was the basis for 15 studies, followed by apps for head and neck cancer patients (*n* = 10), lung cancer patients (*n* = 3), breast cancer patients (*n* = 2), esophageal cancer patients (*n* = 1), and prostate cancer patients (*n* = 1). Two studies focused on palliative patients [11, 12], while the majority set a focus on definitive or adjuvant RT/RCT in a curative setting. Most of the studies were designed as single-arm studies testing the feasibility of a certain app or were only descriptive in nature (*n* = 26). Seven studies had a two-arm design (mostly randomized) [13–19].

**Table 1:** Results of the literature search, apps for patients (ePROs only)

Author, year	App function (name of app or study)	Trial type	Patients included	Patient collective	Conclusion	Commercially available in app stores
Buergy et al. 2019 [20]	Monitoring side effects in follow-up (EORTC questionnaires; MyOnCare, TeleGraPH study)	Single arm	29	Geriatric cancer patients^b^	App-based follow-up might be possible in highly selected elderly patients with modest compliance rates (individual compliance rate: 58.3%)	Yes (iOS and Android)
Cox et al. 2011 [11]	Monitoring symptoms (daily and weekly scales, overnight submission to HCP; HealthHUB, CareHub)	Single arm	21	Patients receiving palliative RT to lung cancer	HCP acknowledged potential benefits of incorporating computerized patient assessment from both a patient and an HCP perspective	Yes (iOS and Android)
Di et al. 2017 [15]	Tracking side effects/QoL, re-examination reminder, knowledge base, and online expert (interactive) during follow-up (6 months)	Prospective, two arms	65/67	Nasopharyngeal cancer receiving RCT	App can improve exercise compliance, reduce adverse reactions and complications (lower incidence of mucositis, xerostomia, and mouth-opening difficulties)	No
El Shafie et al. 2018 [21]	ePROs (general performance, QoL [EORTC QLQ C30], symptoms, need to consult a physician [interaction possible] during RT; OPTIMISE-1)	Single arm	50	Patients with thoracic and pelvic tumors receiving curative RT	Study protocol only	No
Falchook et al. 2015 [31]	Monitoring patient reported outcomes daily during treatment (fatigue, pain, nausea, anxiety), possible interaction with HCP	Single arm	22	Head and neck cancer patients receiving RT (curative)	High compliance and high satisfaction	No
Friedman et al. 2016 [38]	Real-time symptom management during RT with alert system (reminder to enter pain level four times a day; Prime Health MD, Dunwoody, USA)	Single arm	24	Head and neck cancer with definitive RCT (curative)	Feasible app for monitoring pain and severe symptoms in head and neck cancer patients during RT	No^d^
Gani et al. 2019^a^ [33]	ePROs during and after RT	Single arm	23	Patients receiving RT/RCT (curative)	High acceptance (80% weekly feedback), potential to optimize patient care	No
Hauth et al. 2019 [34]	Scoring side effects (CTCAE) and QoL during and after RT (weekly; PROMetheus)	Single arm	21	Mixed cohort of patients receiving RT/RCT (curative)	Successful implementation of an ePRO system, high patient acceptance	No
Hecht et al. 2022^a^ [63]	ePROs during follow-up (twice weekly, 1–10 scale; Patienta, developed by Alcalta, Erlangen, Germany)	Single arm	25	Ambulant oncologic patients^b^	Early detection of possible deterioration of health status possible	No
Kessel et al. 2018 [22]	QoL (EORTC QLQ C 30) during and after treatment	Single arm	81	Mixed cohort at radiation oncology department	Usability test showed good results regarding attractiveness, operability, and understandability. High overall acceptance	No
Maguire et al. 2015 [35]	Symptom monitoring during RT with correspondence to HCP handset (ASyMS)	Single arm	16	Patients with lung cancer receiving RT (curative)	Feasible and acceptable in clinical practice	No
Moller et al. 2022 [32]	ePRO (QoL) during and 4 weeks after RT (MyHospital, developed by MedWare, Odense, Denmark)	Single arm	40	Prostate and cervical cancer receiving pelvic RT (curative)	Feasible, high adherence to weekly self-reporting, time spent acceptable	No
Rades et al. 2020 [14] and 2022 [65]	Assessment of patient-reported outcome (symptoms of pneumonitis, QoL, other adverse effects) to detect a radiation pneumonitis during and after RT (PARALUC)	Prospective, single arm	57	Lung cancer patients receiving RT/RCT (curative)^c^	Patient satisfaction with score and app (prototype) was very high. The developed score to detect pneumonitis showed excellent diagnostic accuracy	No
Sprave et al. 2020 [18]	Monitoring and support app during RT (daily questions for symptoms, QoL, need for personal physician appointment; APCOT study)	Randomized, two arms	100	Head and neck cancer patients receiving RT/RCT (curative)	Study protocol only	No
Sundberg et al. 2021 [19], Sundberg et al. 2017 [67] Sundberg et al. 2015 [68] Langius-Eklöf et al. 2017 [69]	Reporting and managing symptoms during and 3 months after RT (real-time submission to HCP; Interaktor)	Non-randomized two arms	64/66 17/28	Prostate cancer patients undergoing RT	Less symptom burden at the end of treatment in emotional functioning, insomnia and urinary-related symptoms with the app. Increase of patients’ sense of security and their reflections on their own wellbeing	No
Teckie et al. 2021 [37]	ePROs in follow-up 8 weeks after RT (biweekly questionnaires) (LogPAL developed by Northwell Health Inc, Lake Success, USA)	Single arm	38	Head and neck cancer patients receiving RT/RCT (curative)	Feasible, regularly used, and accepted (73.2% questionnaires completed)	Yes (iOS)
Underwood et al. 2022 [39]	Assessment of patient-related outcome after RT (“say all your symptoms” and symptom tracking CTCAE; mPROS app)	Single arm	25	Patients receiving RT to head and neck, breast and pelvic areas	Usable and feasible tailored assessment for patients to report symptomatic toxicities	No
Wöller et al. 2022^a^ [46]	Follow-up app (Myoncare app, Oncare)	Single arm	38	Prostate and breast cancer receiving radiotherapy (curative)	For breast cancer patients: Interest in new communication with HCP (preliminary results)	Yes (iOS and Andoid)
Wong et al. 2018 [24]	Oral mucositis pain assessment (visual analog scale 1–10, reminder 4 ×/day) and accelerometer (activity monitor) to track physical activity	Single arm	Not stated	Head and neck cancer patients receiving RT (at least 50 Gy; curative)	Study protocol only	No
Zini et al. 2019 [36]	Reporting clinical parameters, quality of life, and symptoms during and after RT	Single arm	10	Head and neck cancer patients receiving RCT	Feasible and acceptable by both patients and oncologists	No

*RT* radiotherapy, *RCT* radiochemotherapy, *ePRO* electronic patient-reported outcome, *HCP* health care professional, *QoL* quality of life, *EORTC* European Organisation for Research and Treatment of Cancer, *CTCAE* Common Terminology Criteria for Adverse Effects

^a^Abstract only

^b^Number of patients with RT not presented

^c^Some patients only used a paper version of the app

^d^Not available in the original version at the time of analysis

**Table 2:** Results of the literature search, apps for patients (diverse topics, ePROs only not included)

Author, year	App function (name of app or study)	Trial type	Patients included	Patient collective	Conclusion	Commercially available in app stores
Birkhoff et al. 2018 [29]	Multipurpose tool: appointment calendar, medication tracker, symptom tracker durin (Health Storylines)	Single arm	32	Adult patients receiving RT	Usable and acceptable, more customization is needed to increase usability	No
Boeke et al. 2022 [23]	Activity tracker during RT and 4 weeks afterwards (GIROfit)	Single arm	23	Patients receiving RT/RCT (curative)	High acceptance. Observed changes in physical activities correlated with patient-reported side effects and QoL in some patients	No
Da Cruz et al. 2021 [30]	Multipurpose tool: reminder/scheduling/symptom tracking/information app during RT (AMOR Mama)	Descriptive (pre-study)	–	Breast cancer patients undergoing RT (planned)	App prototype was considered adequate after been having improved by suggestions of HCP. No patients involved so far	No
Fridstedt et al. 2021 [16]	Digital information tool before, during, and after RT (guided tour, maps, telephone numbers, animated films; Digi-Do)	Randomized two-arms	80/80	Breast cancer patients receiving RT (curative)	Study protocol only	Yes (iOS)
Kauppinen et al. 2019^a^ [28]	Daily scheduling app (HMS, Health Care Mobile Solution)	Single arm	30	Patients receiving RT	Effective tool, high usability	No
Ladbury et al. 2021 [26]	Assistance during RT (educational resource; Oncpatient)	Single arm	20	Patients undergoing RT (curative)	Study protocol only	No
Liao et al. 2022 [17]	Multipurpose tool (knowledge database, interactive online consultation, data upload module), follow-up up to 6 months after RCT	Randomized two-arms	57/57	Nasopharyngeal cancer patients undergoing RCT (curative)	Significantly reduction of side effects with app	No
Pavic et al. 2019 [12], Theile et al. 2017 [64]	Activity monitoring and pain/QoL (sensor-equipped bracelet and app) 12 weeks in follow-up	Single arm	30	Palliative cancer patients^b^	Remote monitoring of health care status in palliative cancer patients is feasible, mostly positive feedback (bracelet was worn in 53%, smartphone was used in 85% of the study)	No
Rades et al. 2020 [13] and 2022 [66]	Reminder app for skin care (4 times daily; RAREST-02)	Prospective, two arms	25/28	Head and neck cancer patients receiving RCT (curative)	The reminder app was associated with significantly less grade ≥ 2 dermatitis in patients receiving RCT and nonsignificantly less grade ≥ 2 dermatitis and mucositis (grade ≥ 2 and ≥ 3) in the entire cohort	No
Starmer et al. 2017 [25]	Support adherence to swallowing therapy during RT (exercise videos, educational content, reminder, interaction with HCP possible; Vibrent)	Single arm	36	Head and neck cancer patients undergoing RT (curative)	App is feasibly integrated into patient care practices. It may assist patients in adhering to treatment recommendations and facilitate communication	No
Stephenson et al. 2018 [27]	Teaching platform, tour of institute, interactive games (Proton U)	Descriptive	0	Pediatric cancer patients receiving proton therapy (curative)	Successful implementation of mobile app for pediatric cancer patients	Yes (iOS)
Yang et al. 2021 [47]	Coaching app that interactively provides online advice about food intake, exercise, and weight changes during and after RCT, additional walk step count (Noom Inc)	Single arm	38	Esophageal cancer patients receiving neoadjuvant RCT	App can help nutritional self-care with less decrease in prognostic nutritional index but no prevention of excessive muscle loss	Yes (iOS and Android)

*RT* radiotherapy, *RCT* radiochemotherapy, *ePRO* electronic patient-reported outcome, *HCP* health care professional, *QoL* quality of life, *EORTC* European Organisation for Research and Treatment of Cancer, *CTCAE* Common Terminology Criteria for Adverse Effects

^a^Abstract only

^b^Number of patients with RT not presented

**Table 3:** Results of the scientific literature search, apps for health care professionals (HCP)

Author, year	App function (name of app or study)	Trial type	Patients included	Patient collective	Conclusion	Commercially available in app stores
Ataei et al. 2020 [40]	Radiotherapy-related calculations (Android only)	Descriptive	–	–	Facilitates radiotherapy physicists’ tasks	No
Gerard et al. 2022 [44]	Resource for self-directed learning in radiation oncology (audio lessons, quizzes, and cases; Rad Onc handbook)	Descriptive	–	–	Easy to use, relevant, knowledge has increased, enhanced learning in 80% of participants	No
Jermoumi et al. 2015 [41]	Modeling RT with in situ dose painting (RAID App, Matlab)	Descriptive	–	–	Potential for subsequent development to guide dose-painting treatment planning using high‑z nanoparticles for different LDR brachytherapy sources and low-keV x‑rays	No
Schiefer et al. 2015 [42]	Measurement of isocenter path characteristics of the gantry rotation with an app	Descriptive	–	–	Mechanical isocenter of the gantry and its path can be defined very rapidly, precisely, and cost effectively	No
Tsang et al. 2015 [45]	Dose calculation (RBApp)	Descriptive	–	–	Users were satisfied. Tool is used for both clinical decision making and educational purposes	Yes (Android)
Wu et al. 2017 [43]	Semiautomatic segmentation of glioma on mobile devices for doctors	Descriptive	129	Glioma patients	Comparison with other segmentation methods demonstrates both efficiency and stability of the proposed approach	No

*RT* radiotherapy, *keV* kilo-electron volt

Most patient-centered apps (*n* = 22) focused on ePROs during or/and after RT. This included recording of all types of RT-related side effects and symptoms like pain, general performance, and quality of life. Sometimes validated questionnaires like the EORTC (European Organisation for Research and Treatment of Cancer) QLQ C30 were used [13, 14, 20–22]; in other studies, this was not stated in detail. Side effects were usually evaluated concerning the irradiated area, e.g., swallowing difficulties, dryness of mouth, skin reaction, and mucositis in head and neck cancer patients. The time period of ePRO data collection was either during RT (*n* = 12), during follow-up (*n* = 8), or in both periods (*n* = 10).

Although not clearly stated in every publication, in seven ePRO-centered studies, the possibility of direct contact with a specialized HCP was provided beyond the general contact form.

Three apps supplied an activity tracker [12, 23, 24]. Two reminder apps for head and neck cancer patients were found, one with the goal of completing skin care four times a day [13] and one to support adherence to swallowing therapy during RT [25]. Three apps supplied information on therapy (films, references) [16, 26], one study especially for pediatric patients receiving proton therapy [27]. One study gave assistance in scheduling appointments [28]. Three apps supplied multipurpose features including collection of ePRO as well as an appointment calendar, reminder, and/or knowledge databases [17, 29, 30].

As described in Tables 1 and 2 in detail, most patient-centered studies were single-arm studies or descriptive in nature. Overall, those studies showed high compliance [20, 31, 32], high acceptance [22, 23, 29, 33–37], effectiveness [28], satisfaction [31], usability [29, 38, 39], simplicity [38], and feasibility [12, 25, 32, 35–37, 39]. The randomized two-arm studies analyzing the impact of an app on the standard of care showed a significant reduction in adverse effects in head and neck cancer patients [13, 15, 17] and prostate cancer patients undergoing RT [19]. Five studies did not provide any results because they have so far only published study protocols [16, 18, 21, 24, 26].

The six apps designed for HCP focused on the following topics: RT-related calculations for physicists (*n* = 3) [40–42], semiautomatic segmentation tool for gliomas (*n* = 1) [43], resources for self-directed learning for trainees (*n* = 1) [44], and dose calculations (*n* = 1) [45]. All apps for HCP were descriptive in nature and showed good feasibility, in particular “easy to use” [44], “time efficient, accurate, and simple” [42], “user satisfaction” [45], and “efficient” [43].

### Search in app marketplaces

Twenty apps were found in iOS App Store (Apple) and a total of 13 in Google’s Play Store. Seven apps could be found simultaneously in both stores. In a total of 26 apps, four were designed for patients (workflow management, *n* = 1; guidance of breath-hold management, *n* = 1; information on RT, *n* = 2). One app was specifically dedicated to prostate cancer patients in need of RT. In contrast, 22 apps were designed for HCP. Those can be divided into BED (biological effective dose)/EQD2 (2-Gy equivalent dose) calculators (*n* = 10), reference tools/e-journals (*n* = 5), learning tools for students/young professionals (*n* = 4), physics calculations (*n* = 1), and patient/workflow management (*n* = 2). Most of the apps were free of charge; only two apps for HCP ranged between 0.99 € and 2.99 €. Five apps disclosed in-app purchases. In summary, all identified apps had a maximum of one review (in App Store) and had been downloaded > 100–5000 times (Play Store). Only one app had been downloaded > 10,000 times.

**Fig. 3:**
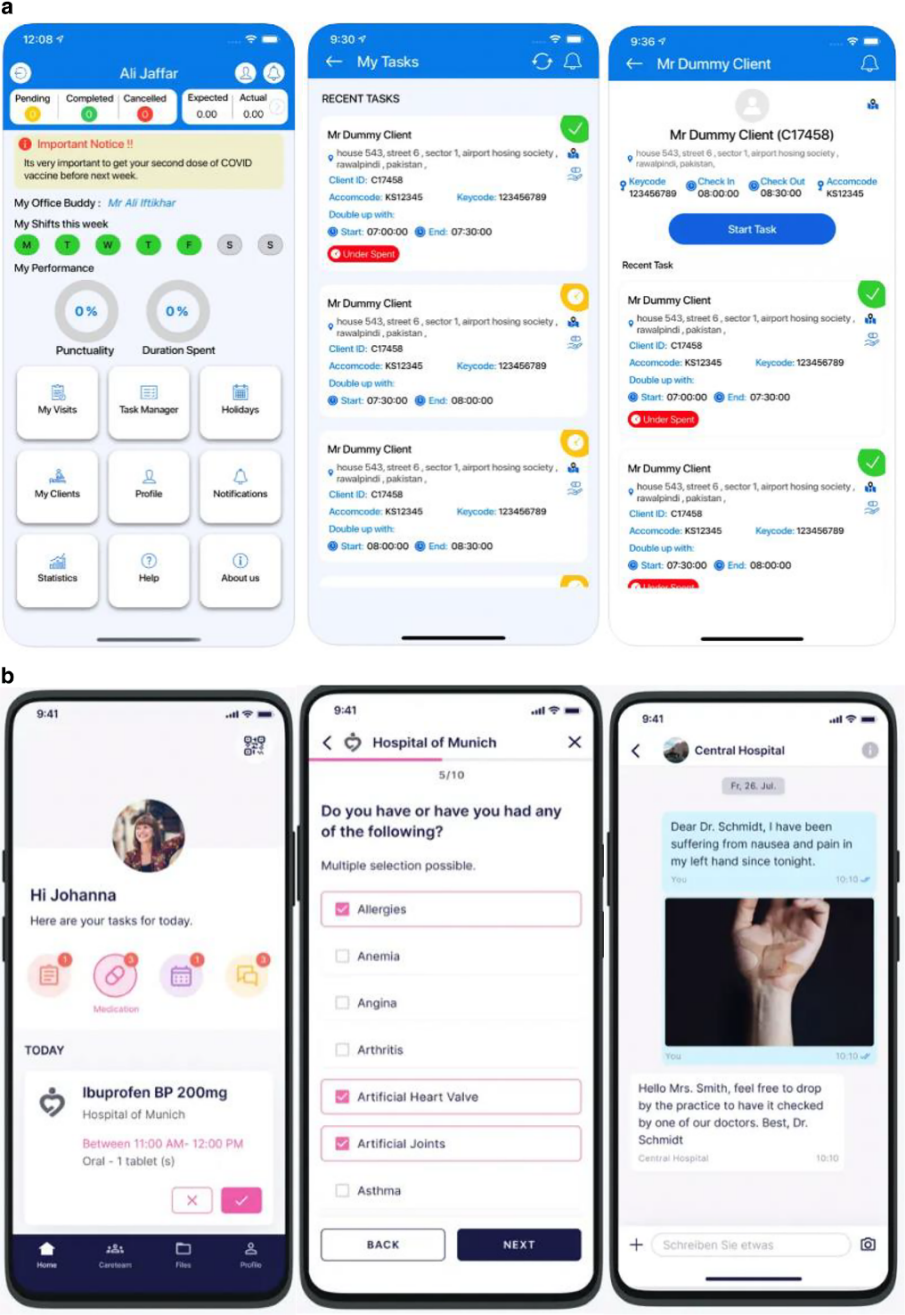
**a** Example of a representative app for digital care management for health care professionals and patients: Care Hub Mobile App (Care Hub Digital Limited, v3.3.4, via App Store). Developed in the UK. App download and use free of charge. Password-restricted access. Features: monitoring of medication and task changes. Automation of assessments. Use not restricted to radiotherapy. Evaluation results: no user reviews available so far. **b** Example of a representative app for digital therapy and health care assistance: MyOnCare App (ONCARE GmbH, v1.6.2, via App Store). Developed in Germany. App download and use free of charge. Password-restricted access (via QR code of participating medical center). Features: exchange of medical data, (e.g., medication plans, support, activities, etc.) between health care professionals and patients. Use not restricted to radiotherapy. Evaluation results: 5 out of 5 (12 user reviews)

Of all scientific publications included in the first part of this review, seven featured apps (one app was included in two independent studies) were also available in the two major app stores (18%) [11, 16, 20, 27, 37, 45–47]. Three of them were designed specifically for the field of radiation oncology (one dose calculation app [45] and two information apps [16, 27]), while four apps did not have a direct reference to radiation oncology but were tested in a patient collective receiving RT (two digital health management apps [20, 37, 46], one symptom tracker [11], and one body weight management app [47]). Figure 3 shows two representative examples of apps included in this review found in Apple’s App store.

## Discussion

Along with the increase in smartphone usage in all age groups, there is a growing interest in mobile health apps. Both HCP and patients are in favor of the use of oncological apps to support treatment adherence, to monitor side effects, and to improve communication [6, 7]. A body of research has been published focused on apps in the general/clinical oncological setting [5]. RT is an innovative, future-oriented discipline [48], representing a major pillar in cancer treatment and requiring special considerations. To our knowledge, this is the first review article to give a comprehensive overview of existing scientific literature on radiation oncology apps and the availability of commercial RT apps in major digital marketplaces.

In comparison to clinical oncology [5], our search revealed a limited number of research papers focusing on apps in radiation oncology. Of those, most studies consisted of a one-arm testing of a certain app or were descriptive in nature. Still, the vast majority of single-arm studies showed feasibility, high acceptance, and patient satisfaction. In the prospective two-arm studies with published results, apps showed improved exercise compliance in follow-up, with reduced adverse reactions for head and neck cancer [13, 15, 17] and increased compliance/communication in patients with prostate cancer [19] compared to the regular standard of care without the use of an additional app. However, the positive impact was largely limited to improving symptom control/reducing side effects. These results are in line with a study of Osborn et al. reviewing mHealth apps of patients with cancer (no special focus on RT). Their overview article included 17 studies with smartphone apps or internet portals collecting data on symptoms or patient activity. In summary, they showed statistically significant differences in ePROs when symptom monitoring using an app was compared to usual care [49]. The authors concluded that apps might improve aspects of symptom control in patients with cancer, but there was only little evidence for impact on other outcomes (e.g., mortality, cancer-related morbidity, long-term outcomes) [49]. Another systemic review of studies on symptom management interventions in people with advanced cancer also showed web- and mobile-based interventions to be efficient in decreasing the overall physical symptom burden [50].

ePROs, as seen in the majority of articles analyzed in our review, are still collected in a generally uncoordinated fashion. In recent years, the European Organisation for Research and Treatment of Cancer (EORTC) has tried to provide guidance in terms of ePRO measurement [51]. However, the problem of unstandardized measurements has already been stated by Giordano et al. [52]. In comparison to laboratory data, there is no common terminology or a common standard for accessing and analyzing ePROs [52]. This is in line with our results showing a wide variety of ePRO measurements in different studies. Mostly, evaluation focused on possible symptoms according to the RT field, but no standard was defined. This also applies to the times of PRO measurements: some study groups focused on the time period during RT (ranging from multiple daily to once-a-week collections), while others gathered data exclusively after RT (during follow-up), or both.

In addition to their importance in clinical day-to-day life, the collection of ePROs or other data (e.g., blood pressure, heart rate, etc.) via apps might also be useful and convenient in randomized controlled trials (so called smartRCTs), especially in the field of radiation oncology [53]. While legal limitations concerning data protection might be a hurdle, apps could potentially reduce costs, study duration, and subjectivity bias [53]. However, to our knowledge, there are currently no existing smartRCTs in the radiation oncology research field.

Collection of app-based ePROs could also hold some disadvantages: Hauth et al. stated that strategies to handle the large amount of data are among the major challenges to be addressed in the future [34]. Moreover, in an early paper published in 2011, Cox et al. pointed out some concerns particularly for palliative patients, where personal contact and the intuition of experienced HCP play an important role. Some clinicians found the age of patients often too advanced, with concomitant rapid deterioration of their condition, to be able to genuinely participate in a study using e‑technology [11]. Still, we found several examples of encouraging results in elderly patient cohorts. Buergy et al. found app-based follow-up feasible in a patient group ≥ 60 years [20]. In the randomized study of Sundberg et al., median age was 69 years [19]. Mean ages of around 60 years were described by others [13, 31, 39]. In line with the demographic transformation and an increase in personal usage of smartphones in the age group ≥ 65 years [54], we believe that the elderly population benefits from technical innovations and should not be excluded.

However, one still has to take the concerns of Cox et al. about personal contact und its impact into account [11]. Nevertheless, the use of new technologies does not automatically imply the neglect of personal contact. On the contrary, apps can be an additional tool to enhance patient empowerment and to personalize the patient–HCP relationship. Future app developers should therefore aspire to these goals.

In 2019, Cunha et al. published a review of the literature on mobile apps for remote support of RT patients similar to our review and found only four articles in the English language [55]. We revealed significantly more publications (*n* = 38). Apart from the fact that our search was more comprehensive, this shows that the existing literature is increasing, reflecting interest in this topic. More than half of the articles found in our search had been published within the past 3 years.

In the second part of our review, we searched digital marketplaces for available radiation oncology apps. A similar attempt was carried out by the study group of Charbonneau et al. [9]. They included 123 apps for general oncology: 50% of apps focused on general information for cancer, followed by specific apps for breast cancer (15%) and skin cancer (7%). Interactive features, including the ability to monitor symptoms, side effects, and treatment, were found in 20% [9]. These observations were similar to the RT-specific results presented here. However, we identified more apps for head and neck cancer patients, which is probably due to the fact that these patients often receive RT/RCT. Lu et al. found 41 apps, with the majority (73%) being general health/pain symptom trackers, when searching app stores for oncological apps [56], which also confirms our findings. Another review article focused on the more specific topic of apps in radiation oncology and was therefore more comparable to our search [57]. In total, Calero et al. identified 31 apps. However, the study group also included tumor staging apps which are not specific to radiation oncology. After subtracting these apps, 13 apps which would have met our inclusion criteria remained. The fact that we identified 39 apps in our search further underscores the emerging interest in this topic in the past few years.

Interestingly, we found a great discrepancy between our search results in scientific literature and search results for existing apps in the two major app marketplaces. While the literature search mainly returned apps for patients (ePROs, information, reminder, and workflow management), the two major app stores contained only four patient-centered apps (three for information/teaching and one workflow management). Only for one app found in app stores was a corresponding scientific publication found. On the other hand, 18% of scientific research articles dealt with an app that was available commercially. Most of those apps were not specifically designed for radiation oncology, but rather general health care apps tested in a radiation oncology setting. This was also seen in the review of Ana, where several apps tended not to be available after completion of the studies [5]. One reason for this discrepancy could be legal difficulties in setting up patient apps outside a clinical trial, with expensive accreditation procedures, e.g., CE marking or accreditation to the European Device Regulations (EU MDR), and reservations on data safety as well as an obligation to update information [58, 59]. Amortization of development, validation, and accreditation costs of RT apps may be facilitated by reimbursement frameworks with prescriptions for RT apps by the healthcare provider [59]. However, the potential target group for radiation oncology apps might be too small to compensate accreditation and maintenance costs. Nonetheless, the demand for digital education in radiooncology has increased since the beginning of the COVID-19 pandemic, especially among medical students [60–62]. In conclusion, while more and more apps are being developed and tested, currently existing apps for HCP and patients found in app stores lack scientific background, whereas clinically validated apps do not become available through app stores or as prescribable medical products.

There are certain limitations to our study. First, in both parts of the review, i.e., the literature search and the app marketplace, search terms for general/clinical oncology were excluded. While beyond the scope of this review, we acknowledge that oncology apps could significantly contribute to advances in radiotherapy, especially for patients undergoing chemotherapy in addition to radiotherapy. Moreover, certain synergistic intersections between oncology and radiotherapy regarding the collection of ePROs (e.g., EORTC QLQ C30 quality of life forms) are conceivable.

Secondly, apps found in our research were downloaded for evaluation but not tested in full detail or even rated.

## Conclusion

The current scientific literature provides some evidence for helpful apps in the field of radiation oncology, with a clear emphasis on apps for patients. The vast majority cover ePROs during or after RT. Most studies are single armed, showing the “feasibility” of tested apps without further randomized testing against standard of care. In contrast, app marketplaces mainly offered apps for dose calculations for HCP in the field of radiation oncology. Therefore, efforts directing the transfer of existing scientific research into the development of commercially available apps in the field of RT should be undertaken.

In pursuing this goal, creation of quality-assured apps for RT patients which combine information sources, ePROs during and after therapy, and the possibility of contact with an HCP would be desirable. Ideally, such apps could transfer highly standardized ePRO data to a hospital interface for easy analysis. Moreover, app development in the field of radiation oncology should involve a certification process of radiation oncology societies, with subsequent testing in trials. After successful studies, apps should be transferred for continuous support of patients and HCP outside the trials [5]. Furthermore, ePROs could also be used for app-accompanied clinical trials (smartRCTs) [53].
